# Galectin-3: A Novel Marker for the Prediction of Stroke Incidence and Clinical Prognosis

**DOI:** 10.1155/2022/2924773

**Published:** 2022-03-01

**Authors:** Ahmed Sayed, Malak Munir, Mohamed S. Attia, Badrah S. Alghamdi, Ghulam Md Ashraf, Eshak I. Bahbah, Mohamed Elfil

**Affiliations:** ^1^Faculty of Medicine, Ain Shams University, Cairo, Egypt; ^2^Department of Pharmaceutics, School of Pharmacy, Zagazig University, Sharkia, Egypt; ^3^Department of Physiology, Neuroscience Unit, Faculty of Medicine, King Abdulaziz University, Jeddah, Saudi Arabia; ^4^Pre-Clinical Research Unit, King Fahd Medical Research Center, King Abdulaziz University, Jeddah, Saudi Arabia; ^5^Department of Medical Laboratory Technology, Faculty of Applied Medical Sciences, King Abdulaziz University, Jeddah, Saudi Arabia; ^6^Faculty of Medicine, Al-Azhar University, Damietta, Egypt; ^7^Department of Neurological Sciences, University of Nebraska Medical Center, Omaha, Nebraska, USA

## Abstract

Stroke, whether ischemic or haemorrhagic, is one of the main causes of mortality and disability all over the world, which entails huge burdens in both healthcare environments as well as social and economic aspects of life. Therefore, there is a continuous search for novel reliable biomarkers that can enhance the recognition of stroke events in a timely manner and predict the clinical outcomes following a stroke event. Galectins are a group of proteins expressed by many types of cells and tissues including vasculature, certain immune cells, fibroblasts, and gastrointestinal epithelial cells. These proteins vary in their structure and configuration according to their type and have a diversity of functions according to the type of tissue they are expressed in. Among these proteins, a few studies investigated mainly the roles played by galectin-1 (Gal-1) and galectin-3 (Gal-3) in the molecular mechanisms of atherosclerosis and in brain tissue remodeling after a stroke event. In this review, we present an updated overview of the current understanding of Gal-3's functions and implications in stroke occurrence and the response of the brain tissue to stroke events, which may be a key to its utility as a predictor of stroke incidence and clinical prognosis in the future.

## 1. Introduction

Stroke is one of the leading causes of disability and mortality worldwide. As defined by the American Heart Association, the term “stroke” encompasses any acute neurological deficit originating from a vascular origin [1]. This definition includes cerebral ischemia, intracerebral haemorrhage, and subarachnoid haemorrhage. Thus, stroke can be classified into two major categories, ischemic stroke —accounting for almost 85% of stroke incidences— and haemorrhagic stroke [1, 2]. Due to the rising incidence rate as well as the significant healthcare, social, and economic burdens of stroke, stroke events have been intensively studied, and several modifiable risk factors of stroke have been identified including hypertension, atherosclerosis, cardiovascular disease, diabetes, and smoking [3].

Despite the fact that patients with such risk factors are considered to be at a higher risk of stroke than the general population, there is still a need for reliable biological markers which can accurately predict the diagnosis of stroke in suspected patients, especially in populations at risk. Additionally, these markers would be of much benefit if they can further predict the clinical prognosis following a stroke event, which typically factors in the management plans of stroke patients. To meet this need and improve the standards of care, several biochemical markers are currently under investigation to assess their potential ability to predict stroke incidence. Of these markers are galectins, a group of glycan-binding proteins known for their role in intracellular interactions and immune regulation. In this literature review, we are discussing the molecular basis of galectins' functions under normal physiological conditions, their utility as predictive and/or prognostic makers following stroke, and how they may alter stroke management.

## 2. Biochemistry of Galectins and Their Physiological Roles

Amongst the many complex glycan-protein interactions which govern numerous intracellular processes, galectins serve a variety of important functions [4]. Galectins are abundantly expressed in many tissues, including gastrointestinal epithelial cells [5, 6], the adventitia of blood vessels [7], fibroblasts [8], thymocytes [9], leukocytes [10], and neuroglial cells [11]. Numerous biological functions are attributed to the expression of galectins including the recognition of cell damage, adherens junction formation, and cell growth regulation. Additionally, they are known to play vital roles in immunological processes including inflammation, apoptosis, phagocytosis, cellular activation, and migration [12].

All galectins share a homologous carbohydrate recognition domain (CRD) which has an affinity to *β*-galactosides, and they can be classified into three subfamilies based on the organization of their CRD: (1) prototypical galectins (Gal-1, 2, 7, 10, 13, and 14) with only one CRD, (2) chimeric galectins (Gal-3) with one CRD and an N-terminal extension, and (3) tandem-repeat galectins (Gal-4, 8, 9, and 12) with two nonidentical CRDs connected by a short peptide chain [4]. As a result of such differences in their structure, galectins can form quaternary structures, with prototypical galectins forming homodimers, tandem-repeat galectins forming dimers, and chimeric galectins forming higher-order oligomers [4]. These different structures determine the physiological functions of the different types of galectins. Currently, 16 galectins have been identified in mammals, 12 of which have been isolated in humans [13, 14] (Table 1). Only six have been identified in the human brain (Gal-1, 2, 3, 8, 9, and 14) [15], with Gal-3 being the most frequently investigated in clinical settings for stroke prediction and prognosis. As a result, this review will focus primarily on the implications of Gal-3 in the pathogenesis and outcome of stroke.

## 3. The Pathophysiological Role of Galectins

### 3.1. The Role of Galectins in the Pathogenesis of Stroke

**Figure 1:**
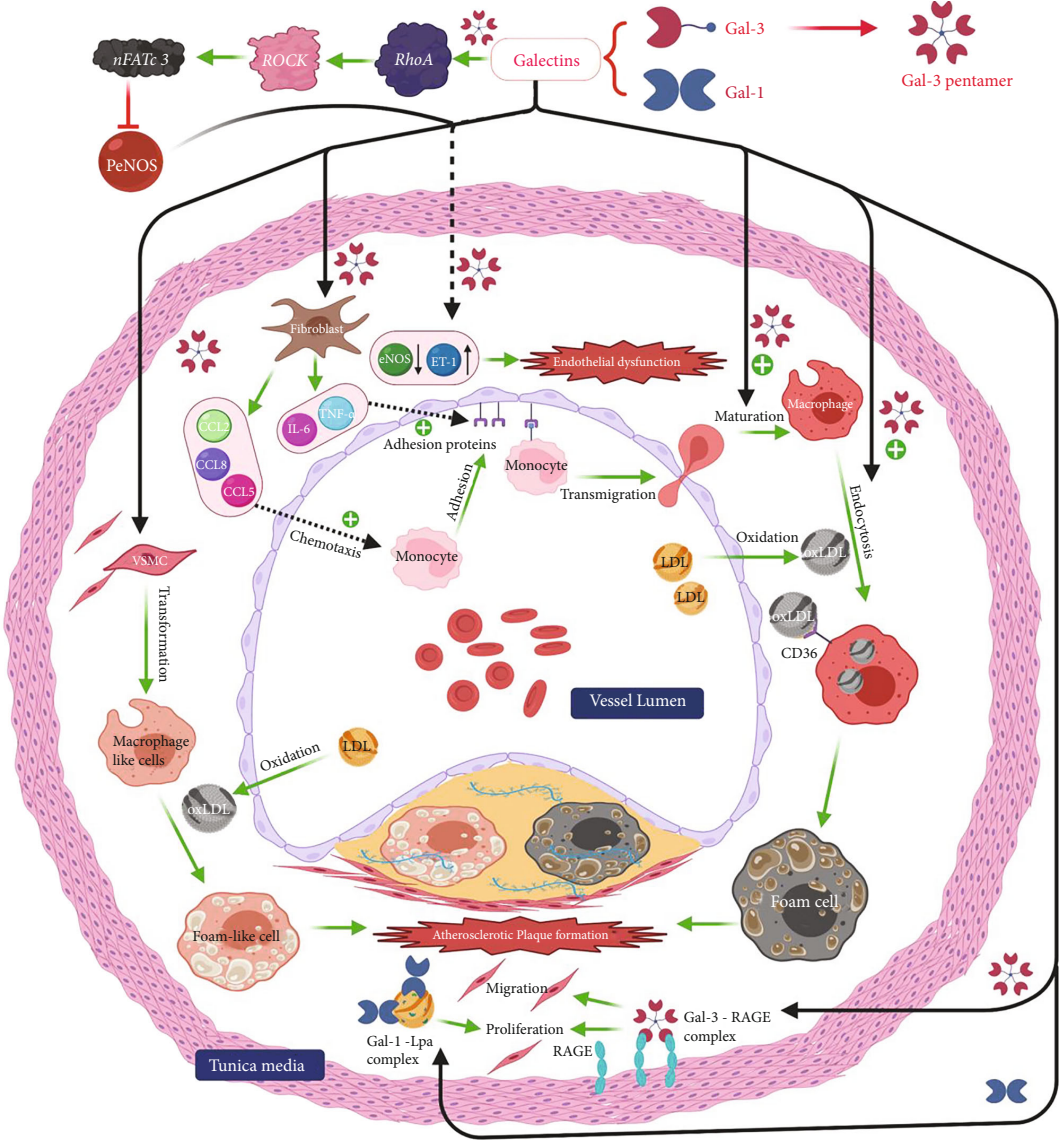
Demonstrates mechanisms and different pathways by which galectins can participate in the pathological process of atheromatous plaque formation as well as the induction of endothelial injury within blood vessels. RhoA: Ras homolog family member A; ROCK: Rho-associated protein kinase; nFATc3: nuclear factor of activated T-cells cytoplasmic 3; LDL: low-density lipoprotein; PeNOS: phospho-endothelial nitric oxide synthase; ET-1: endothelin 1; IL-6: interleukin 6; CD36: cluster of differentiation 36; VSMC: vascular smooth muscle cells; ox-LDL: oxidized LDL; RAGE: receptor for advanced glycation end products; TNF: tumor necrosis factor; CCL 2/5/8: inflammatory chemokine ligands. This figure was created via BioRender (http://www.BioRender.com).

Atherosclerosis is a complex multifactorial pathological process that might lead to an array of cerebrovascular diseases. Over the years, galectins have been shown to be intimately involved in this process. For instance, Gal-1 expression has been positively correlated with the proliferation of smooth muscle cells in specimens obtained from atherosclerotic blood vessels [16]. A potential mechanism of this process is the ability of Gal-1 to bind to lipoprotein (a) (Lp(a)) [17], leading to its accumulation in arterial walls. Lp(a), in turn, triggers smooth muscle proliferation [18, 19] which is a main component of the atherosclerotic process [20] (Figure 1).

There is also evidence of Gal-3's involvement in the atherosclerotic process [21], as shown by its upregulation in human atherosclerotic plaques [22] and the beneficial effects reported in animal studies following inactivation of Gal-3 gene [23]. In apolipoprotein E- (ApoE-) deficient mouse models, when compared with mice with active Gal-3 expression, mice in which Gal-3 gene was knocked out showed a decreased number of the atherosclerotic lesions and reduced amounts of perivascular inflammatory infiltrates [23]. Gal-3 may exert its atherogenic effects via several mechanisms which include mediating the differentiation of monocytes to macrophages and subsequent transformation to foamy macrophages [24, 25]. These foamy macrophages are considered to be the pathological hallmark of atherosclerotic lesions [26]. Moreover, by acting as a chemoattractant when released by foamy macrophages, Gal-3 may enhance the recruitment of more inflammatory cells to the atherosclerotic plaque [27]. Besides, it might be implicated in the transformation of vascular smooth muscle cells into macrophages and the cellular uptake of oxidized low-density lipoproteins (ox-LDL) and advanced glycation end products leading to the production of more foam cells [25]. It can also exacerbate the inflammatory process of atherosclerosis through the activation of the *β*_1_-RhoA-JNK signaling pathway [28], which ultimately aggravates endothelial injury [28].

The intimate association between galectins and the various aspects of atherogenesis, an essential risk factor for cerebrovascular events, is precisely what drove a number of researchers [29–31] to investigate the potential value of measuring galectin levels as a potential method of predicting stroke occurrences and other adverse vascular events.

Furthermore, galectins are known to have immunomodulatory effects, with Gal-3 being the most studied galectin in this regard. Gal-3 is the only chimeric galectin expressed by human cells, suggesting that its quaternary structure might play a role in determining its diverse range of functions. It is ubiquitously produced by various tissues including epithelial cells, cardiac myocytes, vascular endothelial cells [32], leukocytes [33], and glial cells [34]. De Giusti et al. [35] reported upregulation of Gal-3 expression by activated microglia and astrocytes in mice with induced encephalitis, bringing to light the possible involvement of Gal-3 in the pathophysiology of central nervous system (CNS) diseases.

Microglial cells are responsible for the immune reactions in the CNS and respond to brain injury in a graded manner known as microglial activation: a process that involves cell migration and proliferation leading eventually to an inflammatory response with the release of cytokines and other inflammatory markers [36]. In this regard, microglial cells play a dual role in the brain's response to injury; firstly, they are responsible for the release of cytokines that promote cell death. Secondly, they produce trophic factors stimulating cellular proliferation and mediating phagocytosis, both of which are processes that allow for healing without further tissue damage [37, 38]. In addition, Barguillos et al. [39] have shown that the release of Gal-3 by microglia exerts a proinflammatory effect by acting as a ligand for Toll-like receptor 4 (TLR 4), with depletion of Gal-3 attenuating the inflammatory response. The protective effect attained by Gal-3 knockout was also demonstrated by Doverhag et al. [40] in mouse models of hypoxic-ischemic brain injury, whereby Gal-3 deletion reduced oxidative stress, matrix metalloproteinase, and overall brain injury.

**Figure 2:**
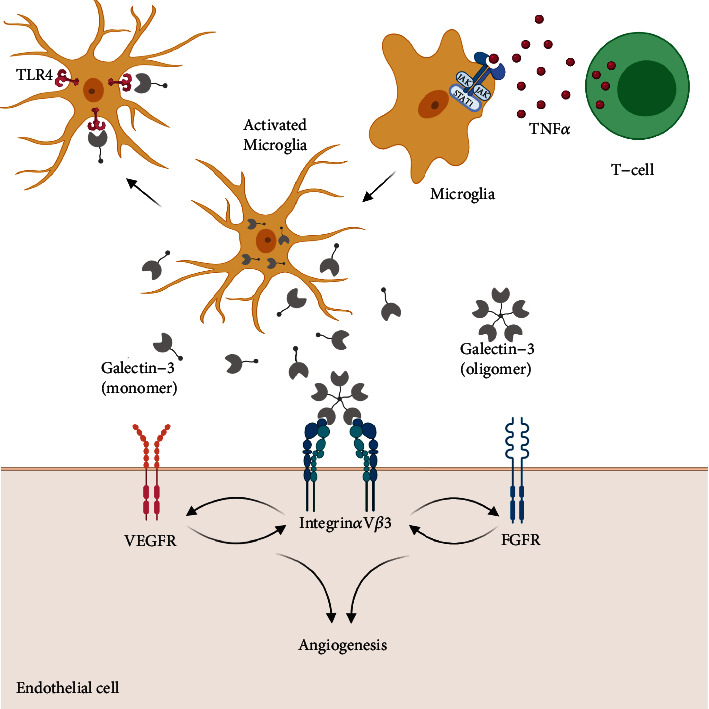
Potential effect of galectin-3 during stroke. TNF*α*: tumor necrosis factor; TLR4: Toll-like receptor 4; VEGF: vascular endothelial growth factor; bFGF: basic fibroblast growth factor. This figure was created via BioRender (http://www.BioRender.com).

On the one hand, Gal-3 is coexpressed alongside insulin growth factor-1 in microglial tissue in response to ischemic injury, where Gal-3 acts not only as an activator of microglia but also as a modulator of injury-induced microglial proliferative response [41]. Gal-3 is secreted extracellularly under the effect of interferon gamma (a proinflammatory cytokine) where it acts as a regulator of microglial and astrocytic response by modulation of JAK-STAT signaling [42] (Figure 2). Such an increase in the expression of Gal-3 was proven to take place during the first few days following a stroke event [43]. Gal-3 deficiency was found to worsen ischemic injury, suggesting that Gal-3 interactions might contribute to the resolution process [41] which might be explained by the loss of Gal-3's regulatory role of microglial activation and proliferation in response to ischemic brain injury. The anti-inflammatory effects of Gal-3 on ischemic lesions were further corroborated when delayed administration of recombinant Gal-3 was found to exert neuroprotective effects as it mediates a shift in the microglial activity away from the proinflammatory subtypes [44].

On the other hand, it was reported that Gal-3 released after acute inflammation, induced by intranigral lipopolysaccharide injection, acts as a Toll-like receptor 4 (TLR4) ligand. By binding to TLR4 on microglial cells, Gal-3 activates microglia into the proinflammatory variant and thus propagates inflammation and exacerbates cell damage [39]. The proinflammatory action of Gal-3, in a different type of injury (brain trauma), was also mediated by Gal-3's ability to bind to TLR4, and administration of antibodies against Gal-3 was found to be neuroprotective in this setting [45]. Dong et al. [46] reported similar findings, where Gal-3 gene knockout reduced the levels of proinflammatory cytokines and had an overall neuroprotective effect in the setting of ischemic brain injury.

In addition to its influence on glial cells, Gal-3 might also be involved in tissue remodeling and angiogenesis following ischemic strokes. Known mediators of angiogenesis and remodeling include matrix metalloproteinases (MMP), angiopoietin, and several growth factors, the most notable of which are vascular endothelial growth factor (VEGF) and basic fibroblast growth factor (bFGF). In vitro studies suggested that Gal-3 acts in a VEGF-dependent manner to promote angiogenesis. It can do this via its chimeric structure. The interaction between the N-terminal CRD on Gal-3 and *α*v*β*3 integrin, which leads to integrin clustering, suggests that Gal-3 can trigger VEGF and bFGF to stimulate angiogenesis via an integrin-dependent signaling pathway [47]. This finding is further supported by Gal-3-induced localization of the VEGF receptor and subsequent increase in VEGF signaling on endothelial cell membranes [48]. Animal studies revealed increased expression of Gal-3 by brain cells following ischemic injury with attenuation of postischemic angiogenesis following the inhibition of Gal-3 [49]. Knocking out Gal-3 gene has also proven to suppress VEGF upregulation following middle cerebral artery occlusion in a mouse model [50], which provides more evidence for VEGF involvement in postischemic angiogenesis induced by Gal-3.

In summary, Gal-3 not only is intimately involved in the atherosclerotic process but also contributes to postischemic tissue remodeling by promoting angiogenesis and healing. It also has a fundamental role in regulating the immune response to acute brain injury. However, the answer to the questions of whether and how it acts to potentiate or to attenuate inflammation is still unclear. Its pleiotropic effects on the immune system coupled with its differential expression and the pathophysiological differences between different types of brain injuries suggest that our current knowledge is somewhat limited and that future studies need to take into account these variables. To that end, although the current lack of understanding of Gal-3's overall role in stroke limits its potential as a therapeutic target, it could still be a valuable biomarker to be used in the management of stroke patients, given that levels of Gal-3 increase significantly in response to acute brain injury.

## 4. The Role of Galectins in Stroke Prediction

### 4.1. Predictive Value in the General Population

Taking into consideration the vital role galectins were shown to play in atherosclerosis, a number of studies have investigated their utility in predicting the incidence of stroke in the general population, due to the well-known relationship between atherosclerosis and stroke. Jagodzinski and colleagues [31], via univariate analysis, initially showed a significant association between the levels of Gal-3 and stroke. However, this association became nonsignificant after adjustment using the Framingham risk factors in multivariate models, suggesting that it was Gal-3's positive association with typical stroke risk factors that drove the initial significant association, rather than a contribution independent of these risk factors. To evaluate the predictive utility of Gal-3, the investigators used three measures: the C-statistic, the integrated discrimination improvement (IDI), and the net reclassification improvement (NRI). Then, they assessed whether adding Gal-3 improved these predictive measures. Disappointingly, both the C-statistic and the IDI showed no significant improvements after adding the value of Gal-3. However, using the continuous NRI, they showed that Gal-3 addition resulted in significant improvements to the previous models, but the categorical NRI with four risk groups showed no such improvement [31].

Another study assessed the value of Gal-3 in improving stroke prediction in the general population through the analysis of the data from the Reasons for Geographic and Racial Differences in Stroke (REGARDS) cohort [29]. In this study, the significant association between Gal-3 and stroke events was no longer seen after adjustment for age, which encouraged the investigators to stratify the study participants by age, where a significant association was seen in the younger (<64 years) but not the older study participants. However, it should be taken into consideration that even in the younger study population where Gal-3 was a more reliable stroke predictor, the association became nonsignificant after adjustment for Framingham risk and socioeconomic factors [29].

The investigators hypothesized that much of the association seen between stroke and galectins may have been due to galectin's association with intermediary inflammatory processes. In the elderly, these processes result into the development of diabetes and hypertension, both of which are disorders with well-known inflammatory components [51, 52]. However, in the younger population, inflammation may not yet have led to the development of hypertension and diabetes. The end result is that in younger populations, the effect of inflammation can only be accounted for by Gal-3, whereas in older populations, where hypertension and diabetes are already more prevalent, Gal-3's independent contribution becomes minimal. Indeed, this would also explain why Gal-3's association —even in the young— was also severely attenuated when adjusting for other typical stroke risk factors, such as hypertension and diabetes. This is consistent with the prior literature, where Gal-3 levels were reported to be significantly increased in diabetic patients as compared with prediabetics, suggesting a positive correlation between plasma glucose levels and the levels of Gal-3 [53]. This might be ultimately suggesting an association between Gal-3 levels and risk factors leading to stroke. An important limitation of the abovementioned studies that may have limited understanding the actual Gal-3's predictive value is that Gal-3 measurements were only done once at baseline and the investigators were not able to explore the value of changing Gal-3 levels and how they might impact future stroke risk.

All in all, Gal-3's predictive role may prove quite important in certain subsets of population, particularly younger individuals, whereas it may be relatively less useful in those with already-established risk factors for stroke. Further research should focus on confirming the utility of Gal-3 as an independent prognostic marker in younger individuals where it might be of most value.

### 4.2. Predictive Value in Populations at High Risk of Stroke

Studies aimed at assessing galectins' utility as a predictive tool for stroke have not been exclusively limited to the general population. Indeed, the utility of galectins in specific populations, known to be at particularly high risk of stroke, may be of a greater value than their utilization in the general population. Edsfeldt et al. [30] investigated the association between increased Gal-3 levels and stroke incidence following carotid endarterectomy (CEA) and found that even after adjusting for a number of important covariates (including demographic data, statin use, and smoking status), high Gal-3 levels maintained a significant association with stroke occurrence, with a hazard ratio (HR) of 4 (95% CI 1.6 to 10.4, *p* = 0.004).

Interestingly, subgroup analysis by gender showed that this association seemed considerably stronger in females (HR of 15.1, 95% CI 1.3 to 172.2, *p* = 0.028), as opposed to males, in whom the association was not significant. This may have critical clinical implications as the benefit of CEA in females has been a subject of debate [54], with a number of studies showing a higher risk of perioperative complications in females [55–57]. This debate is particularly pertinent in asymptomatic women with a lower grade (50-69%) of stenosis, in whom a meta-analysis of the Asymptomatic Carotid Surgery Trial (ACST) and the North American Symptomatic Carotid Endarterectomy Trial (NASCET) demonstrated no significant benefit of CEA [55, 57, 58]. If females with the highest risk of stroke following CEA can be identified by measuring Gal-3 levels, this may hold much promise in terms of allowing physicians to selectively operate on those who are most likely to benefit from therapy. However, several considerations must be taken into account while looking at the abovementioned results. The results of the post hoc subgroup analysis by gender need further verification in the future studies as the reported confidence intervals for females were quite wide (1.3 to 172.2), which emphasizes the need for larger studies through which more precise estimates can be obtained. Moreover, the benefits of measuring Gal-3 levels need to be investigated in clinical trials evaluating whether implementation of routine Gal-3 measurements before CEA improves outcomes for patients in clinical practice.

Gal-3 has also been investigated as a predictive marker in dialysis patients, who are at a significantly higher risk of stroke than the general population due to the vascular alterations induced by uremia [59, 60]. In a cohort of dialysis patients, elevated Gal-3 levels were shown to be significantly associated with a higher risk of stroke [61], but after adjustment for confounding variables, Gal-3 only maintained its significance in relation to the composite endpoint of cardiovascular events including sudden cardiac death and nonfatal myocardial infarction as additional components rather than stroke.

## 5. Galectins as Predictors of Clinical Outcomes following Stroke

### 5.1. Postischemic Stroke

In light of its role in inflammation as well as resolution of acute brain injury, Gal-3 has become a biomarker of interest in the prediction of poststroke clinical outcomes. Both ischemic and haemorrhagic strokes are associated with neuronal damage and significant inflammation. Hence, in accordance with the in vitro studies, it is not surprising to find that Gal-3 levels are elevated in proportion to the severity of stroke [46, 62–64].

Ischemic stroke occurs as a result of decreased perfusion due to arterial stenosis/occlusion causing focal ischemia, hypotension, or increased intracranial pressure leading to global ischemia. Increased Gal-3 levels were reported to be positively correlated with stroke severity estimated by the National Institutes of Health Stroke Scale (NIHSS) score with an *r*-coefficient of 0.87 [46]. In addition, the receiver operating curve (ROC) analysis revealed that Gal-3 was able to predict the functional outcome of stroke at a cut-off value of 53.5, with a sensitivity of 88.4% and a specificity of 76.9% (area under the curve (AUC) = 0.884, 95% CI 0.827 to 0.941, *p* < 0.001), where functional outcomes were categorized into favourable and poor outcomes according to the modified Rankin scale (mRS) (mRS score of 0-2 = favourable outcome; mRS score of 3-6 = poor outcome) [46]. In agreement with these findings, a multivariable adjusted-spline model showed a linear relationship between Gal-3 level and poor clinical outcome following stroke [63]. The authors also reported that Gal-3 was significantly associated with death and/or major disability 3 months postischemic stroke independently of other risk factors [63] (Table 2). On the other hand, a pilot study by Bustamante et al. [65] reported contradicting results suggesting that Gal-3 is not an effective biomarker for prognostic prediction in patients with ischemic stroke. However, this study had a very small sample size of only 26 patients, which limits the conclusion one could draw from this study's results.

### 5.2. Posthaemorrhagic Stroke

Haemorrhagic stroke can be classified into intracerebral and subarachnoid haemorrhage. Despite being less common than ischemic stroke, haemorrhagic stroke is associated with greater morbidity and mortality [66]. Therefore, being able to accurately predict the clinical prognosis following haemorrhagic stroke events could allow for modifying management plans according to the predicted clinical scenarios in order to achieve better outcomes. In a cluster of 110 patients with intracerebral haemorrhage, a multivariate analysis by Yan et al. [64] revealed that a high level of Gal-3 was an independent risk factor for a poor prognosis. Analysis of the ROC showed that the Gal-3 level was successfully used to predict one-week mortality, six-month mortality, and six-month poor outcomes with a moderate sensitivity/specificity at respective cut-off points (see Table 2). In the same cohort, Gal-3 was found to significantly improve the C-statistic of the mainstay predictors of the haemorrhagic stroke outcome (NIHSS score and hematoma volume), but this was only true for long-term outcomes (6-month mortality and poor outcome) [64]. Liu et al. [62] reported similar findings in patients with subarachnoid haemorrhage as elevated Gal-3 levels were an independent risk factor for six-month mortality and poor outcome with a sensitivity/specificity of 77.8%/70.6% and 81.1%/77.1%, respectively (Table 2). Nevertheless, there are discrepancies between the two studies. For example, Yan and colleagues [64] reported that Gal-3 levels were higher in nonsurvivors compared to survivors at 6 months, whereas Liu et al. [62] reported the opposite. It might be the difference in haemorrhagic stroke types between the two studies that is responsible for such discrepancies.

Overall, studies on the prognostic value of Gal-3 in the prediction of stroke outcomes are scarce, and the available data are limited by a number of factors. Firstly, most of the studies were done on relatively small population samples which makes it difficult to apply any of the findings to the general population of stroke patients. Furthermore, most studies showed that Gal-3 levels were only moderately predictive of the stroke outcome. As a result, it is difficult to make a definitive decision as to whether Gal-3 testing is beneficial as a prognostic marker for stroke. Based on that, more prognostic studies on larger population samples are needed.

### 5.3. Modification of Gal-3's Prognostic Effect by Glycaemic Level

Hyperglycaemia is a common presenting symptom of stroke that most likely occurs as a result of the release of cortisol and noradrenaline [67]. The relationship between hyperglycaemia and the efficiency of Gal-3 level, as a prognostic marker in stroke patients, was investigated. Zeng et al. [68] reported on the role of hyperglycaemia in improving the predictive function of Gal-3 levels. In a cohort of 3,082 ischemic stroke patients, hyperglycaemic patients with higher Gal-3 levels (≥10.58 ng/mL) were found to be at greater risk of developing the primary composite outcome (death and vascular events), stroke recurrence, and vascular events only one year after a primary stroke event, with an adjusted HR of 1.72, 2.64, and 2.68, respectively, as opposed to the normoglycaemic cohort in which Gal-3 had a low prognostic value [68]. The mechanism by which hyperglycaemia enhances the prognostic efficacy of Gal-3 is still unclear. Consequently, more molecular studies are needed to better outline the functions and interactions of Gal-3, and this in turn will allow improved clinical utilization of Gal-3 levels in special cohorts.

## 6. Conclusion and Future Recommendations

In summary, the current evidence suggests a potentially important role of galectins in the development and progression of stroke, and their interaction with other variables, such as plasma glucose level, requires further confirmatory studies. There are attempts to implement these findings in clinical practice by investigating the utility of galectins in two primary domains: the prediction of stroke occurrence and the prognosis of stroke.

As to the prediction of stroke, results seem to be promising when applied to specific subpopulations, such as younger individuals and female patients undergoing CEA. This suggests that galectins may have a greater diagnostic value if applied to specific portions of the population. Nevertheless, these results are in need of further confirmation by dedicated studies investigating the utility of galectins in those particular groups. Furthermore, future studies should also investigate the role of serial changes in Gal-3 levels and how they might impact stroke occurrence, as current studies have only investigated the effect of baseline measurements.

When it comes to the clinical prognosis of stroke, galectins have proven potential utility in both ischemic and haemorrhagic strokes, with studies showing an important association between the levels of galectins and clinical outcomes of stroke that is deserving of further study. Future studies integrating Gal-3 measurements in clinical settings may advance the management of patients with this debilitating condition.

## Figures and Tables

**Table 1:** Structure and function of galectins found in humans [14, 69].

Galectin	Subfamily	Function	Main tissue distribution
Gal-1	Prototypical	Regulation of apoptosis Cellular proliferation and differentiation	Nonspecific
Gal-2	Prototypical	Unknown	Gastrointestinal Urogenital
Gal-3	Chimeric	Immunomodulation and recognition of cell membrane damage	Macrophages Fetal membranes Gastrointestinal
Gal-4	Tandem-repeat	Adherens junction formation	Gastrointestinal
Gal-7	Prototypical	Regulation of apoptosis Control of cell growth	Skin Oesophagus
Gal-8	Tandem-repeat	Recognition of cell membrane damage Restricts infection by viral and bacterial pathogens	Nonspecific
Gal-9	Tandem-repeat	Immunomodulation Bactericidal	Gastrointestinal
Gal-10	Prototypical	Immunoregulation Forms Charcot-Leyden crystals in eosinophils	Bone marrow
Gal-12	Tandem-repeat	Regulation of apoptosis	Adipose Breast
Gal-13	Prototypical	Regulation of apoptosis^∗^	Placenta
Gal-14	Prototypical	Regulation of apoptosis^∗^	Placenta
Gal-16	Monomer	Regulation of apoptosis^∗^	Placenta

^∗^Strong regulator of T-cell apoptosis.

**Table 2:** Association of plasma Gal-3 levels with stroke outcomes.

Ischemic stroke					
Dong et al. [46]	Outcome	Sensitivity	Specificity	Cut-off value	
	Functional outcome	88.4%	76.9%	53.5 pg/mL	
Wang et al. [63]	Outcome	Q1^∗^	Q2^∗^	Q3^∗^	Q4^∗^
	Death or major disability (OR)	1.00	1.09	1.16	1.55
	Death (OR)	1.00	0.74	0.81	2.10
	Major disability (OR)	1.00	1.13	1.19	1.43
Haemorrhagic stroke					
Yan et al. ([64])	1-week mortality	73.3%	79.4%	28.9 ng/mL	
	6-month mortality	90.9%	64.6%	22.4 ng/mL	
	6-month unfavourable outcome	89.5%	65.5%	18.9 ng/mL	
Liu et al. ([62])	6-month mortality	77.8%	70.6%	24.6 ng/mL	
	6-month unfavourable outcome	81.1%	77.1%	23.4 ng/mL	

^∗^Wang et al. categorized their study participants into 4 subgroups according to the quartiles of Gal-3 levels with Q4 being the highest quartile and Q1 being the lowest one. OR indicates odds ratio.
